# Enhanced Enrichment of Medaka Ovarian Germline Stem Cells by a Combination of Density Gradient Centrifugation and Differential Plating

**DOI:** 10.3390/biom10111477

**Published:** 2020-10-24

**Authors:** Jun Hyung Ryu, Seung Pyo Gong

**Affiliations:** 1 Department of Fisheries Biology, Pukyong National University, Busan 48513, Korea; lion5299@naver.com; 2 Department of Marine-Biomaterials and Aquaculture, Pukyong National University, Busan 48513, Korea

**Keywords:** ovarian germline stem cells, enrichment, density gradient centrifugation, differential plating, Matrigel, fish

## Abstract

Fish ovarian germline stem cells (OGSCs) have great potential in various biological fields due to their ability to generate large numbers of mature eggs. Therefore, selective enrichment of OGSCs is a prerequisite for successful applications. To determine the optimal conditions for the enrichment of OGSCs from Japanese medaka (*Oryzias latipes*), we evaluated the effects of Percoll density gradient centrifugation (PDGC), differential plating (DP), and a combination of both methods. Based on cell morphology and gene expression of germ cell-specific *Vasa* and OGSC-specific *Nanos2*, we demonstrated that of seven density fractions obtained following PDGC, the 30–35% density fraction contained the highest proportion of OGSCs, and that Matrigel was the most effective biomolecule for the enrichment of *Oryzias latipes* OGSCs by DP in comparison to laminin, fibronectin, gelatin, and poly-l-lysine. Furthermore, we confirmed that PDGC and DP in combination significantly enhanced the efficiency of OGSC enrichment. The enriched cells were able to localize in the gonadal region at a higher efficiency compared to non-enriched ovarian cells when transplanted into the developing larvae. Our approach provides an efficient way to enrich OGSCs without using OGSC-specific surface markers or transgenic strains expressing OGSC-specific reporter proteins.

## 1. Introduction

Unlike the adult mammalian species in which the existence of ovarian germline stem cells (OGSCs) is still controversial [1], adult female fish unquestionably have OGSCs within their ovaries, which was clearly confirmed by a study using Japanese medaka (*Oryzias latipes*) [2]. Fish OGSCs are responsible for the production of a large number of mature eggs throughout the life of fish and thus have the potential to be used in various fields, including developmental biology, transgenic animal production, reproduction, and species conservation, as if primordial germ cells (PGCs) and spermatogonial stem cells (SSCs) have done [3]. Several studies have demonstrated the feasibility of the use of fish OGSCs as a biological resource by producing live offspring through the transplantation of crude or enriched ovarian germ cells [4,5,6] or cultured OGSCs [7] into developing larvae.

In *Oryzias latipes*, OGSCs are located in the ovarian epithelial cell layer between the dorsal ovarian cavity and the ventral stromal compartment where germ cells such as OGSCs, cyst-forming germ cells, and diplotene oocytes are surrounded by *Sox9b*-expressing cells in a structure called the germinal cradle [2]. Although the exact proportion of OGSCs within the ovaries of adult medaka has not been verified yet, it is expected to be small since only 1–5 OGSCs are present in a germinal cradle and the distribution of germinal cradles is restricted to the epithelial cell layer of the ovary [2]. Therefore, the isolation of such a small number of pure OGSCs from the ovary is a key issue that remains to be addressed for the use of these cells for various applications.

Despite the current controversy regarding the existence of mammalian OGSCs, until now, several methods have been employed for OGSC isolation from mammalian ovaries, including surface marker-based, transgenic animal-based, and cellular property-based methods [8]. The first two methods are optimal for the specific isolation of OGSCs. However, specific markers for fish OGSCs have not yet been identified [9], and commercially available transgenic fish expressing reporter proteins governed by an OGSC-specific promoter have yet to be developed. Thus, the only currently available and easily accessible method for fish OGSC isolation is based on a cellular property for which there are two main cell separation methods.

Density gradient centrifugation uses cellular size and density to separate specific cells from crude cell populations [10]. Many types of media, such as Percoll, Ficoll, sucrose, glycerol, dextran, serum albumin, Nycodenz, and Histodenzand, can be used for density gradient centrifugation. Percoll, which consists of colloidal silica particles coated with polyvinylpyrrolidone or silane, is the most frequently used. It is non-toxic and does not adhere to cells due to its coated surface. Percoll allows for the creation of iso-osmotic density gradients within a range of 1.0 to 1.3 g/mL. Thus, the properties of Percoll allow for the separation of cells from crude cell populations via differential cell density under non-toxic conditions [10,11]. Effective and rapid cell separation by density gradient centrifugation can thus be achieved without expensive equipment or specialized techniques [12]. Indeed, several studies addressing fish germline cell isolation have demonstrated the effectiveness of density gradient centrifugation for the enrichment of specific types of germ cells [4,13,14,15]. However, most of the studies explored the isolation of male germline cells, whereas only a few studies reported the isolation of fish ovarian germ cells from Siberian sturgeon [16] and a transgenic zebrafish strain expressing a non-OGSC specific fluorescent protein modulated by the *Vasa* promoter [4]. Therefore, more quantitative data with regards to the use of this method for isolation of fish OGSCs are required.

The differential plating (DP) method for cell separation relies on the adherence property of cells. The majority of vertebrate cells are anchorage-dependent in an in vitro environment; some exceptions include blood cells and tumor cells, which are non-adherent. It is well known that GSCs adhere to substrates loosely, unlike other adherent somatic cells that show a relatively strong attachment to substrates [17,18]. Cell adhesion is altered based on the type of substrate molecules used. A previous report demonstrated that mouse SSCs selectively bind to laminin-coated surfaces [19]. In fish, it has been reported that rainbow trout SSCs were obtained at a purity of >95% by serial DP of testicular cells on gelatin-coated plates, indicating that DP is effective for fish germline cell isolation [18,20].

Several studies have successfully used the two cell separation methods (Percoll density gradient centrifugation (PDGC) and DP) in combination to enrich for fish spermatogonia from crude testicular cell populations [17,21]. However, these studies did not evaluate or describe the enrichment efficiency quantitatively, which has made the effectiveness of the combinatorial use of the two methods ambiguous. Furthermore, this kind of trial has not been performed for fish OGSC enrichment.

In this study, we evaluated the efficiency of the cell separation methods for the enrichment of fish OGSCs. The separation methods were evaluated individually and in combination to identify the best approach for fish OGSC enrichment without the need for OGSC-specific surface markers or transgenic strains expressing OGSC-specific reporter proteins. To achieve our aim, crude ovarian cell populations from adult Japanese medaka (*Oryzias latipes*) were first subjected to PDGC or DP to determine the optimal conditions for each method. The combination of the two optimized methods was then used for the enrichment of *Oryzias latipes* OGSCs. During these procedures, the effects of each experimental treatment on OGSC enrichment were evaluated on the basis of cellular morphology and gene expression levels of OGSC-specific *Nanos2* and germ cell-specific *Vasa*. Although several studies have previously used *Vasa* gene expression as a parameter to evaluate GSC enrichment [4,9,20], *Vasa* is a germ cell marker expressed in all types of germ cells from GSCs to gametes [22] and thus cannot provide accurate data for GSC enrichment. By contrast, *Nanos2* is expressed only in OGSCs [2] and we, therefore, speculated that the use of *Nanos2* as well as *Vasa* to evaluate OGSC enrichment could provide more reliable data than the use of *Vasa* alone. Finally, a transplantation assay was carried out to validate the capacity of the enriched OGSCs to localize in the gonadal region of developing larvae.

## 2. Materials and Methods

### 2.1. Animals

Adult Japanese medaka (*Oryzias latipes*) was purchased from a local aquarium and reared in 20 L tanks at 26 °C. Fish were fed two or three times a day with a commercial diet for flounder larvae (EWHA, Busan, Korea) and the photoperiod was maintained in light for 14 h and darkness for 10 h. Prior to experiments, adult females were separated from the others and starved for at least 24 h. All experiments using fish were performed in compliance with ethical guidelines from the Institutional Animal Care and Use Committee (IACUC) of Pukyong National University, which approved our research proposal (approval number: 2016-07).

### 2.2. Tissue Collection and Cell Dissociation

Ovaries were surgically removed from 5 to 10 adult females according to each experiment after anesthesia with 0.1% (*v/v*) 2-phenoxyethanol (Sigma-Aldrich, St. Louis, MO, USA). The ovaries were washed twice with Dulbecco’s phosphate-buffered saline (DPBS; Gibco, Grand Island, NY, USA) containing 1% (*v/v*) penicillin and streptomycin (P/S; Gibco, Grand Island, NY, USA) and dissociated mechanically with sterile scissors. Subsequently, an enzymatic dissociation was conducted in Leibovitz’s L-15 medium (L15; Gibco, Grand Island, NY, USA) supplemented with 500 U/mL collagenase type I (Worthington Biochemical Corporation, Lakewood Township, NJ, USA) for 1 h at 28 °C. After dissociation, an equal volume of L15 containing 10% (*v/v*) fetal bovine serum (FBS; Gibco, Grand Island, NY, USA) was added to the cell suspension and it was filtered through a 40 µm cell strainer (Falcon, Durham, NC, USA) to remove debris. Finally, the cells were washed twice with DPBS containing 1% (*v/v*) P/S and then used for experiments.

### 2.3. Embryonic Cell Culture

Embryonic cells were prepared as described previously [23]. Briefly, an embryo at stage 32 to 36 was disinfected in 70% (*v/v*) ethanol (SK Chemicals, Sungnam, Korea), and the chorion and yolk of the embryo were removed with a syringe needle. Subsequently, an embryo was transferred into a well of 0.1% (*w/v*) gelatin (Sigma-Aldrich, St. Louis, MO, USA)-coated 96-well culture plate (Thermo Scientific, Vernon Hills, IL, USA) and then dissociated using a syringe with 26-gauge needle. Dissociated embryonic tissues were cultured in L15 supplemented with 20% (*v/v*) FBS and 1% (*v/v*) P/S at 28 °C, and passaged at a ratio of 1:2 or 1:3. The embryonic cells at passage 30 were used for experiments.

### 2.4. Percoll Density Gradient Centrifugation (PDGC)

Singly dissociated ovarian cells were suspended in 200 µL DPBS and loaded onto the top of a discontinuous 7-step Percoll (Sigma-Aldrich, St. Louis, MO, USA) solution consisting of 1 mL each of 20%, 25%, 30%, 35%, 40%, 50%, and 60% in a 15 mL conical tube (Falcon, Durham, NC, USA). Each Percoll solution was prepared by the manufacturer’s instruction. After centrifugation at 800× *g* for 30 min, the cells from each density fraction were carefully harvested, washed twice with DPBS, and then used for experiments. Cell morphologies were observed under a phase-contrast inverted microscope (TS-100F, Nikon, Tokyo, Japan). Morphology of OGSCs was defined as cells harboring a large nucleus with one or two prominent nucleoli [24,25].

### 2.5. Differential Plating (DP)

In order to perform DP, five biomolecules including fibronectin (Gibco, Grand Island, NY, USA), laminin (Gibco, Grand Island, NY, USA), Matrigel (Corning Life Sciences, Bedford, MA, USA), gelatin, or poly-l-lysine (Sigma-Aldrich, St. Louis, MO, USA) were used for coating 35 mm Petri dishes (SPL Life Sciences, Pocheon, Korea). For coating fibronectin or laminin, Petri dishes were treated with 20 μg/mL fibronectin dissolved in DPBS or 20 μg/mL laminin dissolved in DPBS containing Ca^2+^ and Mg^2+^ (Gibco, Grand Island, NY, USA) at 37 °C. After overnight incubation, the dishes were washed three times with DPBS and subsequently treated with 0.5 mg/mL bovine serum albumin (BSA; Sigma-Aldrich, St. Louis, MO, USA) dissolved in DPBS at 37 °C for 1 h to prevent non-specific binding. After washing three times with DPBS, the dishes were used for DP. Matrigel coating was performed by treating the dishes with the Matrigel, which was diluted in cold DPBS 10 times, at room temperature for 1 h and subsequent steps were conducted identically with those of fibronectin and laminin. In the case of gelatin, the dishes were covered with 0.1% (*w/v*) gelatin dissolved in distilled water and incubated at room temperature for 2 h. After removing the solution, the dishes were air-dried completely and then used for the experiments. For poly-l-lysine, the dishes were treated with poly-l-lysine, which was diluted in distilled water 5 times, at room temperature for 5 min. After that, the dishes were washed three times with distilled water, air-dried, and used for the experiments. For the separation of cells depending on adhesiveness, the cells dissociated from the ovaries from five adult females were suspended in L15 supplemented with 10% (*v/v*) FBS and 1% (*v/v*) P/S, and 2 × 10^6^ cells were seeded on a 35 mm petri dish coated with one of five biomolecules described above. After overnight incubation at 28 °C, the cells that were floating or loosely adhered to substrates were harvested by washing the dishes with DPBS five times. The remaining cells adhered to substrates were collected after trypsinization with 0.05% trypsin-EDTA solution (Gibco, Grand Island, NY, USA). All harvested cells were washed with DPBS and then used for analyses.

### 2.6. Quantitative Reverse Transcriptase Polymerase Chain Reaction (qRT-PCR) Analysis

For qRT-PCR analysis, the samples (enriched ovarian cells) were prepared from 10, 5, and 10 adult females by PDGC, DP, and a combination of both, respectively. The total RNA of the samples was extracted using RNeasy Plus Micro Kit (Qiagen, Valencia, CA, USA) according to the manufacturer’s instructions. Then, 200 ng of RNA was treated with DNase I (Sigma-Aldrich, St. Louis, MO, USA) and reverse-transcribed with GoScript reverse transcription system (Promega, Madison, WI, USA). Synthesized complementary DNA (cDNA) was subjected to qRT-PCR analysis using LightCycler 480 II Real-Time PCR System (Roche Applied Science, Mannheim, Germany) with SYBR Green I Master (Roche Applied Science, Mannheim, Germany) and specific primers. The primer sequences were listed in Table 1. The mRNA level of each gene in the samples was normalized to that of *β-actin* and calculated by 2^−ΔΔCt^ method, where Ct = threshold cycle for target amplification, ΔCt = Ct_target gene_ − Ct_internal reference (*β-actin*)_, and ΔΔCT = ΔCt_sample_ − ΔCt_calibrator_.

### 2.7. Cell labeling and Transplantation Assay

In these experiments, the ovarian cells from 10 adult females were enriched by a combination of PDGC and DP and then used for cell labeling and transplantation. Cell labeling was performed with a red fluorescent dye, PHK26 (Sigma-Aldrich, St. Louis, MO, USA). To determine the optimal working concentration of PKH26, cells were stained with 0, 2, 4, 6, 8, and 10 µM PKH26 according to the manufacturer’s instruction, and then labeling efficiency and cell viability were measured. For labeling efficiency, the number of labeled cells was counted using a hemocytometer (Marienfeld, Lauda-Konigshofen, Germany) under a TS-100F microscope equipped with a fluorescent unit (Nikon, Tokyo, Japan) and its percentage of the total number of cells was calculated. For cell viability, trypan blue (Gibco, Grand Island, NY, USA) exclusion assay was performed and the cell survival rate was calculated as follows: the number of unstained cells / total number of cells × 100. For cell transplantation, approximately 1000 live cells labeled with PKH26 were transplanted into the peritoneal cavity of recipient *Oryzias latipes* larvae at 11 days post fertilization (dpf) using glass capillaries with pore sizes of 30–50 µm. Anesthesia of recipient larvae was performed with 0.1% (*v/v*) 2-phenoxyethanol (Sigma-Aldrich, St. Louis, MO, USA). After that, the recipient larvae were reared in tanks until 20 dpf, and then the colony formation derived from the transplanted cells near the gonads of developing recipient larvae was examined under a TS-100F microscope equipped with a fluorescent unit. For detecting exact cellular localization, the merged pictures in Figure 6 and Supplementary Figure S3 were taken under both halogen lamp-derived light and UV light filtered with fluorescence filter cube for detecting red fluorescence from PKH26.

### 2.8. Statistical Analysis

The statistical analysis for the data from qRT-PCR was performed using SPSS version 18 (IBM-SPSS, Chicago, IL, USA). The data were analyzed by one-way analysis of variance (ANOVA) or t-test followed by Duncan’s method. Statistical analysis for the data from the transplantation assay was performed using a generalized linear model in Statistical Analysis System software (SAS Institute, Cary, NC, USA). When ANOVA in the SAS package detected a significant main effect, the least-squares method was conducted. Significant differences among groups were determined when the *p* < 0.05.

## 3. Results

### 3.1. Separation of Dissociated Ovarian Cells by PDGC

**Figure 1:**
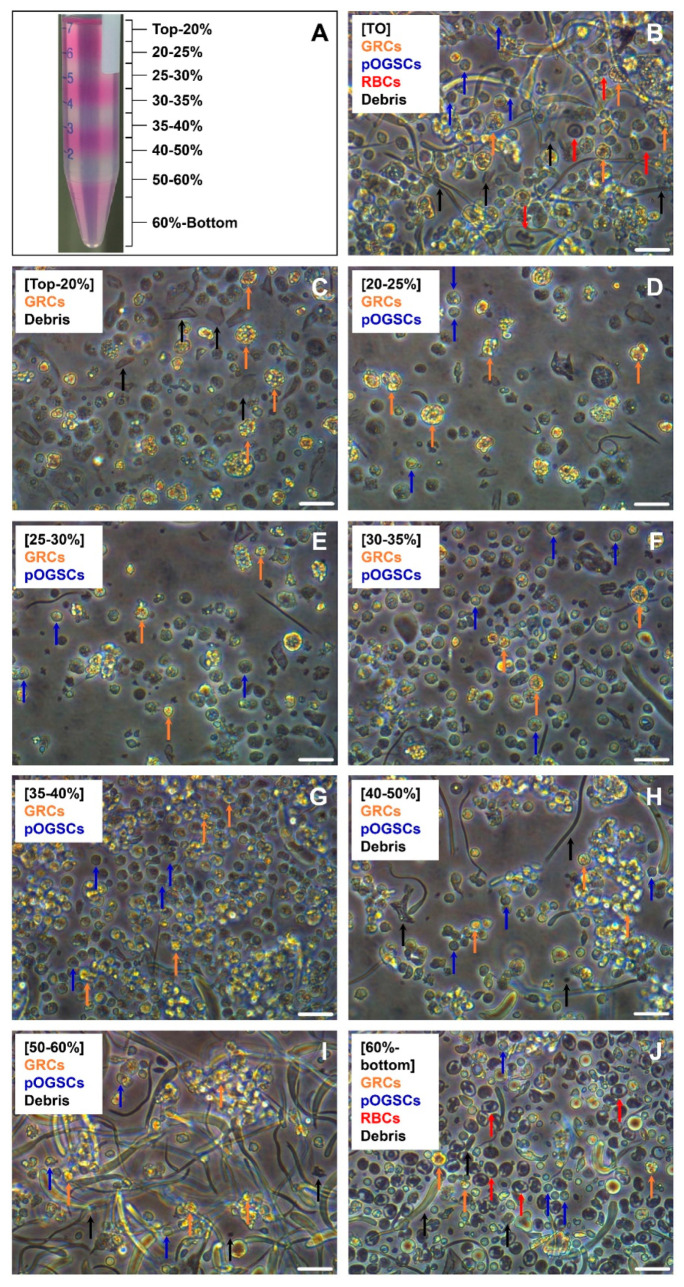
Separation of medaka (*Oryzias latipes*) crude ovarian cell populations by Percoll density gradient centrifugation (PDGC). Ovarian cells obtained from 10 adult females by mechanical and enzymatic dissociation were subjected to PDGC to separate the cells depending on their density. (**A**) Picture taken after PDGC. Density fractions from the top–20%, 20–25%, 25–30%, 30–35%, 35–40%, 40–50%, 50–60%, and 60%–bottom were collected separately, and the cells in each fraction were observed with an inverted microscope. (**B**) Image of the crude total ovarian cell population (TO) before cell separation. Granule-rich cells (GRCs), putative ovarian germline stem cells (pOGSCs), red blood cells (RBCs), and debris were observed. (**C–J**) Images of the cells from each density fraction after PDGC. pOGSCs were most abundant in 30-35% density fraction, and a lot of GRCs were identified in 35–60% density fractions. RBCs were concentrated in the 60%-bottom fraction, and tissue debris was abundant in the top–20% and 50–bottom fractions. Scale bar = 20 µm.

**Figure 2:**
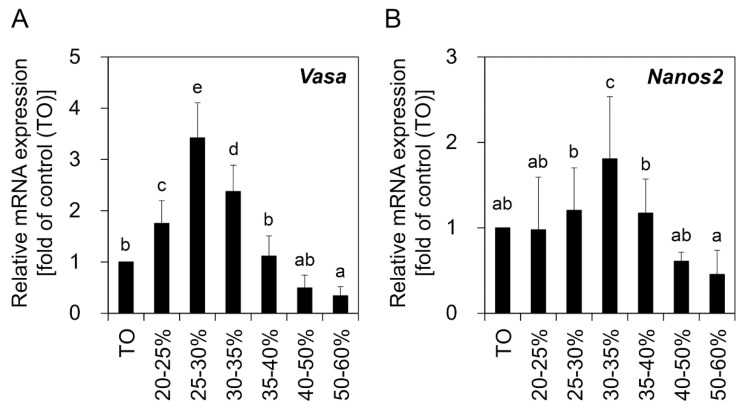
Relative expression of *Vasa* and *Nanos2* genes in the ovary-derived cell populations separated by PDGC. Crude TO was harvested by enzymatic dissociation of the ovaries derived from 10 adult females. Following PDGC, cells from each density fraction were subjected to qRT-PCR analysis for comparison of *Vasa* (**A**) and *Nanos2* (**B**) expression levels. TO was used as a control. Significant increases in *Vasa* expression were observed in the cells from the 20–35% density fractions. For *Nanos2*, the highest expression level was observed in cells from the 30–35% density fraction. The values are expressed as mean ±SD. ^a–e^ Different letters indicate significant differences; *p* < 0.05.

To determine the optimal conditions for OGSC enrichment, the dissociated ovarian cells were separated by PDGC, and the cells harvested from the density fractions were compared in terms of morphology and gene expression. As shown in Figure 1, total dissociated ovarian cells (TO) not subjected to PDGC consisted of several cell types, including putative OGSCs, granule-rich cells (GRCs), and red blood cells (RBCs), in addition to cellular debris. Following PDGC, putative OGSCs were observed in a wide range of density fractions from 20% to 60%. GRCs were mostly observed in the 35–60% fractions, while RBCs were concentrated in the 60%–bottom density fraction and tissue debris was abundant in the top-20% and 50%-bottom density fractions. qRT-PCR analysis of germ cell-specific *Vasa* and OGSC-specific *Nanos2* revealed that the expression levels were significantly higher in cells from the 20–35% and 30–35% density fractions for *Vasa* and *Nanos2*, respectively, compared to TO (Figure 2, *p* < 0.05). Additionally, when one-to-one comparisons of the results were performed by t-test, the cells from the 40–60% density fractions showed significantly lower expression levels compared to TO for both *Vasa* and *Nanos2* (*p* < 0.05). Based on these results, the 20–40% density fractions were selected for subsequent experiments.

### 3.2. Effects of Different Adhesion Biomolecules in DP on OGSC Enrichment

To find the most effective adhesion biomolecules for DP of OGSCs, the effects of five adhesion biomolecules, including laminin, fibronectin, Matrigel, gelatin, and poly-l-lysine, were tested. Dissociated ovarian cells were incubated overnight in plates coated with each of the five adhesion biomolecules. Adherent and non-adherent cells from each of the plates were collected and subjected to qRT-PCR analysis to compare levels of expression of *Vasa* and *Nanos2*.

**Figure 3:**
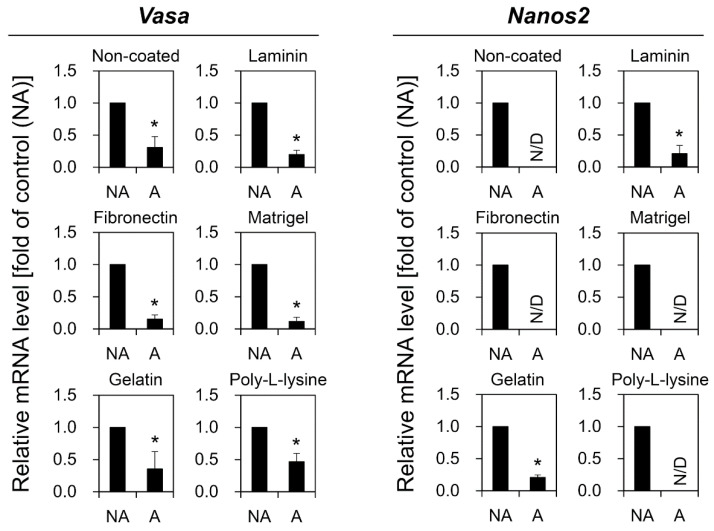
Comparison of *Vasa* and *Nanos2* expression between non-adhesive (NA) and adhesive (A) cells after differential plating (DP). Crude TO from five adult females was subjected to DP on plates coated with different adhesion molecules including gelatin, fibronectin, laminin, Matrigel, and poly-l-lysine. NA and A cells were collected separately and analyzed by qRT-PCR to compare *Vasa* and *Nanos2* expression levels. Regardless of the type of adhesion molecule used, the expression levels of both *Vasa* and *Nanos2* were significantly higher in NA than A cells. The values are expressed as mean ± SD. Asterisks (*) indicate significant differences; *p* < 0.05. N/D, not detected.

**Figure 4:**
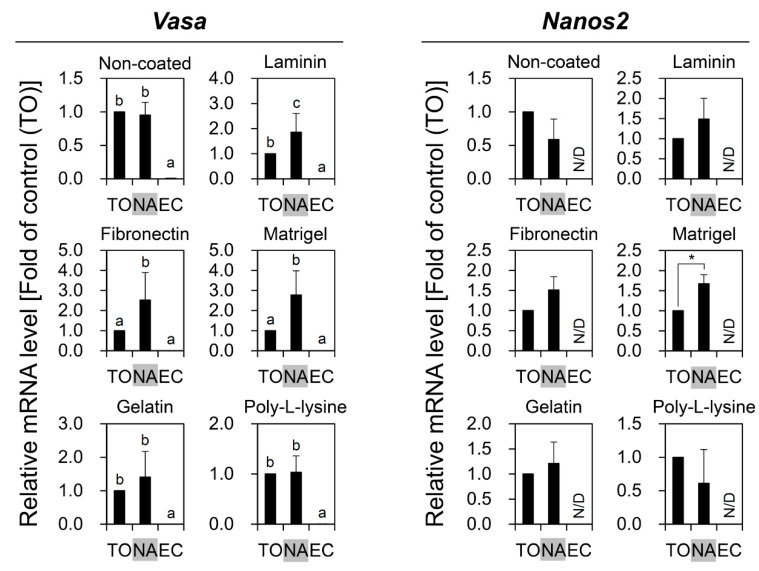
Comparison of *Vasa* and *Nanos2* expression between crude TO and NA cells derived from DP of TO. TO from five adult female fish was subjected to DP on plates coated with gelatin, fibronectin, laminin, Matrigel, or poly-l-lysine. NA cells were then collected from each plate. TO, NA, and Japanese medaka embryonic cells (EC), which were used as a negative control, were subjected to qRT-PCR for comparison of *Vasa* and *Nanos2* expression levels. Significant increases in *Vasa* expression compared to TO were observed in NA cells from laminin, fibronectin, or Matrigel-coated plates. Only NA cells from Matrigel-coated plates showed significantly higher *Nanos2* expression than TO. The values are expressed as mean ±SD. ^a-c^ Different letters and asterisk (*) indicate significant differences; *p* < 0.05. N/D, not detected.

Significant differences were observed in terms of cell adhesion. Plates coated with gelatin, fibronectin, laminin, or Matrigel showed a high proportion of adhered cells, whereas only a few cells adhered to poly-l-lysine or uncoated plates (Supplementary Figure S1). The harvested cell populations of adherent and non-adherent cells showed different gene expression levels of both *Vasa* and *Nanos2*. For *Vasa*, non-adherent cells showed significantly higher gene expression levels regardless of the type of adhesion molecules used (Figure 3). For *Nanos2*, no expression was detected in the adherent cells retrieved from the uncoated, fibronectin-, Matrigel-, or poly-l-lysine-coated plates, whereas *Nanos2* expression was detected in the non-adherent cells from the corresponding plates. In laminin- or gelatin-coated plates, significantly higher *Nanos2* expression was observed in the non-adherent cells than in the adherent cells (Figure 3). These data indicate that the OGSCs were mainly included in the non-adherent cells rather than adherent cells after DP. Next, the expression of *Vasa* and *Nanos2* in the non-adherent cells derived from each plate was compared with TO and embryonic cell (EC) lines to evaluate the effectiveness of each type of adhesion molecule on OGSC enrichment. As shown in Figure 4, *Vasa* expression was significantly increased in the non-adherent cells from plates coated with laminin, fibronectin, Matrigel, or gelatin relative to TO, but *Nanos2* expression was significantly increased only in the non-adherent cells from Matrigel-coated plates compared to TO. In the ECs used as a negative control, decreased or no expression was observed for *Vasa* and *Nanos2*, respectively. These data confirm that Matrigel is the most effective for the enrichment of OGSCs by DP.

### 3.3. Combinatorial Effects of PDGC and DP on OGSC Enrichment

**Figure 5:**
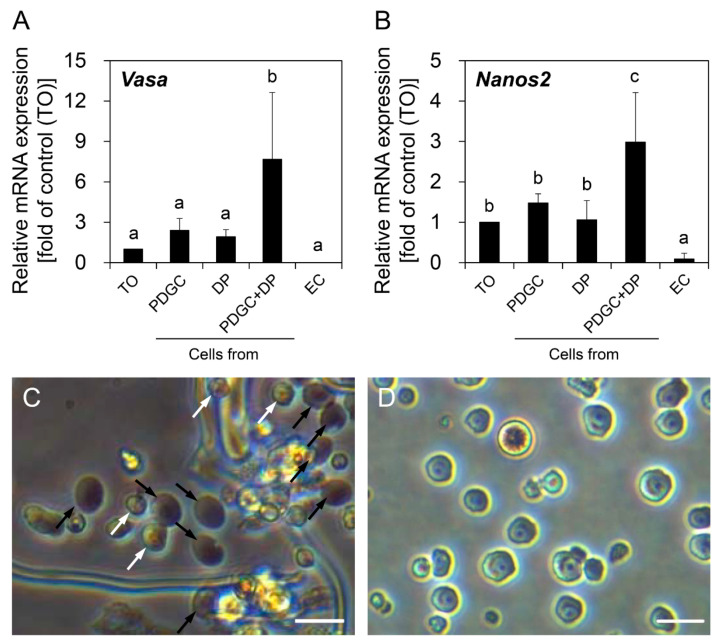
Effects of combinatorial use of PDGC and DP on the enrichment of OGSCs. qRT-PCR analysis for *Vasa* (**A**) and *Nanos2* (**B**) genes to evaluate the enrichment effect of the combinatorial use of PDGC and DP at the gene expression level. Crude TO was obtained from 10 adult females and separated by PDGC, Matrigel-based DP, or a combination of both. Cells from the 20–40% density fractions after PDGC and non-adherent cells after DP were used for analysis. In the combination of PDGC and DP, cells from the 20–40% density fractions after PDGC were subjected to Matrigel-based DP and the final non-adherent cells were used for analysis. Expression levels of *Vasa* and *Nanos2* were significantly higher in the cells separated by PDGC and DP in combination compared to TO and the cells separated by PDGC or DP alone. *Oryzias latipes* EC were used as negative control. The values are expressed as mean ±SD. ^a–c^ Different letters indicate significant differences; *p* < 0.05. (**C**) Morphology of the cells separated by only DP. Putative OGSCs (white arrowheads) and red blood cells (black arrowheads) were observed with tissue debris. (**D**) Morphology of the cells separated by PDGC and DP in combination. Remarkable enrichment of putative OGSCs was observed. Scale bars = 20 µm.

In the next set of experiments, PDGC and DP were used for OGSC enrichment to evaluate if there was a synergistic effect by the combination of both methods. To this end, cells from the 20–40% density fractions after PDGC were subsequently subjected to DP on Matrigel. qRT-PCR analysis showed that cells enriched by PDGC and DP showed significant increases in both *Vasa* and *Nanos2* expression levels compared to TO (Figure 5A,B), indicating that the combination of PDGC and DP had a synergistic effect on OGSC enrichment. The morphological observation indicated that the combination of PDGC and DP enables putative OGSCs to be enriched more effectively than by DP alone, with the additional advantage of the removal of RBCs and debris (Figure 5C,D).

### 3.4. Localization of Enriched OGSCs in Gonadal Region of Developing Larvae

**Figure 6:**
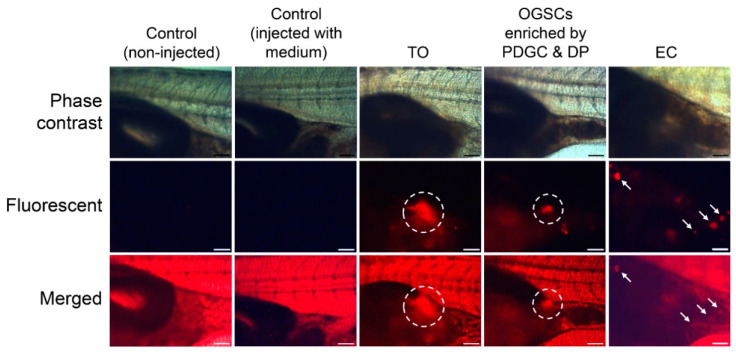
Transplantation assay of OGSCs. Crude TO was obtained from 10 adult females and enriched by PDGC and DP in combination. TO, enriched OGSCs, or *Oryzias latipes* EC were transplanted into a peritoneal cavity of larvae at 11 dpf after labeling with PKH26. Localization of the donor cells in the gonadal region of the recipient larvae was examined at 20 dpf (9 days post-transplantation). The localization of the cells (dotted circles) was observed in the recipient larvae transplanted with TO and the cells enriched by PDGC and DP. The recipients transplanted with EC showed ectopic fluorescent signals (arrows) scattered in posterior abdominal regions. Scale bar = 100 µm.

Prior to the transplantation assay, the optimal PKH26 concentration for cell labeling was determined. Ovarian cells enriched by PDGC and DP in combination were stained with different concentrations of PKH26 and evaluated for labeling efficiency and cell viability. A significant increase in the percentage of fluorescent cells was observed at concentrations higher than 4 µM PKH26, while cell viability was significantly decreased at concentrations higher than 8 µM PKH26, indicating 4–6 µM was optimal for cell labeling (Supplementary Figure S2). To confirm the identity of OGSCs enriched by PDGC and DP in combination, a transplantation assay was performed after 6 µM PKH26 labeling. The survival rates of the recipient larvae transplanted with medium, TO, enriched OGSCs, and ECs at 20 days post fertilization (dpf) ranged from 57.1% to 70.0%. There was no statistical difference compared with control larvae not subjected to transplantation (72.4%) (Table 2). As shown in Figure 6, TO and enriched OGSCs showed colonization near the gonadal region of the recipient after transplantation, while the transplanted ECs showed ectopic localization and the cells dispersed in the posterior abdominal region (for wide-angle pictures of Figure 6, see Supplementary Figure S3). A significant difference in the rates of localization in the gonadal region of developing larvae was detected between TO and the enriched OGSCs. Nine of 44 (20.5%) recipient larvae transplanted with the enriched OGSCs showed localization in the gonadal region, while only 4 of 40 (10%) recipients showed localization when transplanted with TO (Table 2). Collectively, these data suggest that the combination of PDGC and DP effectively enriched OGSCs.

## 4. Discussion

In this study, we demonstrated that the combination of two well-known cell separation methods, PDGC and DP, significantly enhanced the efficiency of OGSC enrichment in Japanese medaka. For quantitative assessment of enrichment efficiency of OGSCs, we used two germline cell-specific genes, *Vasa* and *Nanos2*. Apart from these two genes, it has been known that there are many of genes that are specifically expressed in ovarian germline cells, which includes *Dazl* [26], *Dnd* [27], *Nanog* [28], *Oct4* [29], *Piwi* [30], and *Tdrd1* [31]. In *Oryzias latipes*, it has been revealed that all these genes except *Nanog* are expressed in all stages of ovarian germline cells from oogonia to mature oocytes. However, *Dnd* is expressed more abundantly in oogonia and early-stage oocytes compared to larger oocytes [26] and in case of *Nanog*, it is expressed in OGSCs at a high level but reduced in small oocytes [28]. Therefore, the appropriate combination of these genes as well as *Vasa* and *Nanos2* can be used in the study targeting ovarian germline cells like this by considering the expressional characteristics of each gene.

From our results, we could identify differences in the data derived from the comparison of *Vasa* and *Nanos2* expression between the cell populations segregated by cell separation. In the experiments involving OGSC enrichment by PDGC, putative OGSCs were morphologically identified in the 20–60% density fractions after PDGC, and of those, 20–35% fractions showed significantly higher *Vasa* expression than the other fractions with the highest expression in cells from the 25–30% fraction. These results were similar to those reported in a previous study that showed that zebrafish ovarian cells expressing *Vasa* were abundant in the 25–35% density fractions after PDGC [4]. Moreover, it was also similar to the findings shown in studies for SSC enrichment that demonstrated that the proportion of putative SSCs after PDGC was high in the 25–30% and 30–36% density fractions in zebrafish [15] and loach [14], respectively. For *Nanos2*, however, our data showed that the expression was significantly higher in the 30–35% density fraction, indicating that the proper density fraction to isolate a highly pure OGSC population by PDGC might be 30–35% rather than the 25–30% reported by others in which the highest *Vasa* expression was observed. Nevertheless, we used cells from the 20–40% density fractions rather than 30–35% after PDGC for further testing of DP. The reason for selecting the bigger fraction is that the available cell number for analysis and further tests was too small when the cells were retrieved from a narrow range of density fractions. This point made it difficult to use the cells from a single density fraction of 30–35%, in which the highest *Nanos2* expression was observed, for the next series of experiments. Further studies using large fish from which more ovarian cells could be harvested would enable us to better evaluate the effects of *Nanos2*-based enrichment by PDGC. Similarly, a discrepancy in the gene expression pattern between *Vasa* and *Nanos2* was also observed in the experiments using DP, suggesting that the use of *Nanos2* together with *Vasa* can be used to draw more accurate and reliable data for the evaluation of OGSC enrichment. Nevertheless, additional evidence will be needed from other fish species to support the widespread use of *Nanos2* for evaluating OGSC enrichment.

In the experiment of OGSC enrichment using DP, the expression of both *Vasa* and *Nanos2* was significantly higher in non-adherent cells than in adherent cells regardless of the type of adhesion molecules used. This indicates that ovarian germ cells, including OGSCs, are non-adherent or are less adherent to substrates than ovarian somatic cells, which are normally highly adherent. This is consistent with previous reports that demonstrated that adherent gonadal somatic cells could be removed by DP using gelatin for the enrichment of fish SSCs [17,18] and mouse OGSCs [32]. In mice, SSCs were successfully enriched by selective adhesion to a laminin-coated substrate [33,34], and thus, we expected that laminin would show a similar effect on medaka OGSCs. However, our data show that laminin was not suitable for selective adhesion of medaka OGSCs, suggesting that the adhesion property of gonadal cells is largely similar between mouse and medaka, but the specificity of cells to adhesion molecules is different.

Non-adherent cells retrieved from fibronectin-, laminin-, or Matrigel-coated plates showed significantly higher *Vasa* expression than TO, whereas *Nanos2* expression was significantly higher only in non-adherent cells collected from Matrigel-coated plates. In the case of gelatin, which has been frequently used to remove somatic cells in DP for GSC enrichment, only slight increases in *Vasa* and *Nanos2* expression were observed with no statistical difference. These results suggest that fibronectin, laminin, and Matrigel are more effective for removing ovarian somatic cells compared to gelatin. Matrigel is of special interest as it is the most effective extracellular matrix for enriching OGSCs. Its effectiveness is likely due to its components, which include various adhesion molecules such as laminin, collagen IV, heparin sulfate proteoglycans, entactin, and nidogen [35]. These molecules collectively induce the adherence of diverse kinds of ovarian somatic cells more effectively than individual adhesion molecules.

When the two enrichment methods, PDGC and DP, were combined, the final enriched cells showed significantly higher *Vasa* and *Nanos2* expression compared to cells enriched by PDGC or DP alone. This is likely a result of the effective removal of non-germline cells by PDGC, which removed ovarian cells found in the top–20% and 40%–bottom density fractions, and by DP, which removed highly adherent cells. Moreover, the enriched cells showed a 2.25-fold increase in localization of the cells in the gonadal region compared to TO in the transplantation assay. The results regarding transplantation assay showed relatively low efficiencies compared to common transplantation success (ranging from 43% to 80%) using spermatogonia [36,37,38]. Our results are similar to those of Wong et al. [4,7], which showed 16% to 29.9% efficiencies in cell colonization near the gonadal region after OGSC transplantation according to experimental conditions. Meanwhile, some results from other researches dealing with rainbow trout showed relatively high efficiencies from 41.4% to 71.6% after transplantation [5,6]. Previously, a study reported that colonization efficiency after germ cell transplantation was different from the number of cells transplanted [38]. In that experiment, when 300 or 1000 testicular cells were transplanted into the larva at 11 dpf, colonization efficiency was about 10%. This was increased to approximately 60% when more than 3000 cells were transplanted, but the rate was not raised anymore when 10,000 or 30,000 cells were used. Based on these results, the rate of transplantation success can be significantly affected by the number of cells transplanted. In this study, although we intentionally used a small number of cells for transplantation assay to examine the difference between treatment groups more effectively, the transplantation assay using a large number of cells (>10,000 cells) needs to be performed to increase the success rate of transplantation. Collectively, these results imply that the cells enriched by PDGC and DP in combination contained a high proportion of early-stage germ cells including OGSCs. Our findings are supported by reports that SSCs were successfully enriched by a combination of PDGC and DP in Nile tilapia [17], porcine [39], and goat [40].

## 5. Conclusions

Medaka OGSCs could be effectively enriched by a simple combination of PDGC and DP. The use of Matrigel in particular increased the efficiency of OGSC enrichment. As a method that does not use OGSC-specific surface markers and transgenic strains expressing OGSC-specific reporter proteins, this method can be applied to other fish species for which the molecular signature of the gonads is not yet known. The results of this study will provide useful information for the study and use of fish OGSCs for various applications, including biological research, transgenic fish production, species conservation, and aquaculture.

## Figures and Tables

**Table 1:** Primer sequences used in this study.

Genes	Primer Sequences (5′ > 3′)	Product Size (bp)	Accession No.
*Nanos2*	Forward, GGTGCAAACAACTGTGGATG Reverse, CTTGCAGAAGCGGCAGTAAT	262	NM_001160447.1
*Vasa*	Forward, GAGAAGGTTCCGACCACCAG Reverse, AATGGTGTTGGGCAGGTCAA	177	NM_001104676.1
*β-actin*	Forward, CCACCATGTACCCTGGAATC Reverse, GCTGGAAGGTGGACAGAGAG	153	NM_001104808.1

**Table 2:** Rates of localization in the gonadal region after transplantation of OGSCs enriched by PDGC and DP into 11 days post-fertilization (dpf) larvae.

Donors	No. of Recipients Transplanted	No. (%) ^1^ of Recipients Survived to 20 dpf	No. (%) ^1^ of Recipients that Transplanted OGSCs Localized in Gonadal Region
Control (non-injected)	58	42 (72)	0 (0) ^a^
Medium	40	26 (65)	0 (0) ^a^
Total ovarian cells	40	28 (70)	4 (10) ^b^
Enriched OGSCs	44	28 (64)	9 (20) ^c^
Embryonic cells	42	24 (57)	0 (0) ^a^

^1^ Percentage of the number of recipients transplanted. ^a–c^ Different letters indicate significant differences, *p* < 0.05.

## Supplementary Materials

The following are available online at https://www.mdpi.com/2218-273X/10/11/1477/s1, Figure S1: Separation of medaka (*Oryzias latipes*) crude ovarian cell populations by differential plating (DP), Figure S2: Examination of the optimal concentration of PKH26 for labeling the enriched ovarian cells, Figure S3: Wide-angle pictures of Figure 6.
