# Supplementary material for: Enhanced Enrichment of Medaka Ovarian Germline Stem Cells by a Combination of Density Gradient Centrifugation and Differential Plating
Source: Biomolecules. 2020 Oct 24;10(11):1477. doi: 10.3390/biom10111477 (PMC7690863; doi:10.3390/biom10111477)
Supplement: Supplementary file 1 [file biomolecules-10-01477-s001.zip › biomolecules-945392-supplementary_final/Supplementary Figure S1_final.pdf]

Enhanced enrichment of medaka ovarian germline stem cells by a combination of density gradient centrifugation and differential plating

Jun Hyung Ryu, Seung Pyo Gong

Supplementary Figure S1. Separation of medaka (*Oryzias latipes*) crude ovarian cell populations by differential plating (DP). The crude total ovarian cell population (TO) was harvested by enzymatic dissociation of the ovaries derived from 5 adult females and subjected to DP on the dishes coated with different adhesion molecules including gelatin, fibronectin, laminin, Matrigel or poly-L-lysine. Then, the pictures of the cells after DP were taken to compare cell adherence among the dishes coated with different biomolecules. A number of cells were adhered to gelatin-, fibronectin-, laminin- or Matrigel-coated dishes but few were adhered to non-coated or poly-L-lysine-coated dishes. After the removal of floating and loosely-bound cells that were expected to include ovarian germline stem cells, red blood cells and others, only adhesive somatic cells were found with attached on the substrates. Scale bar=20 μm.

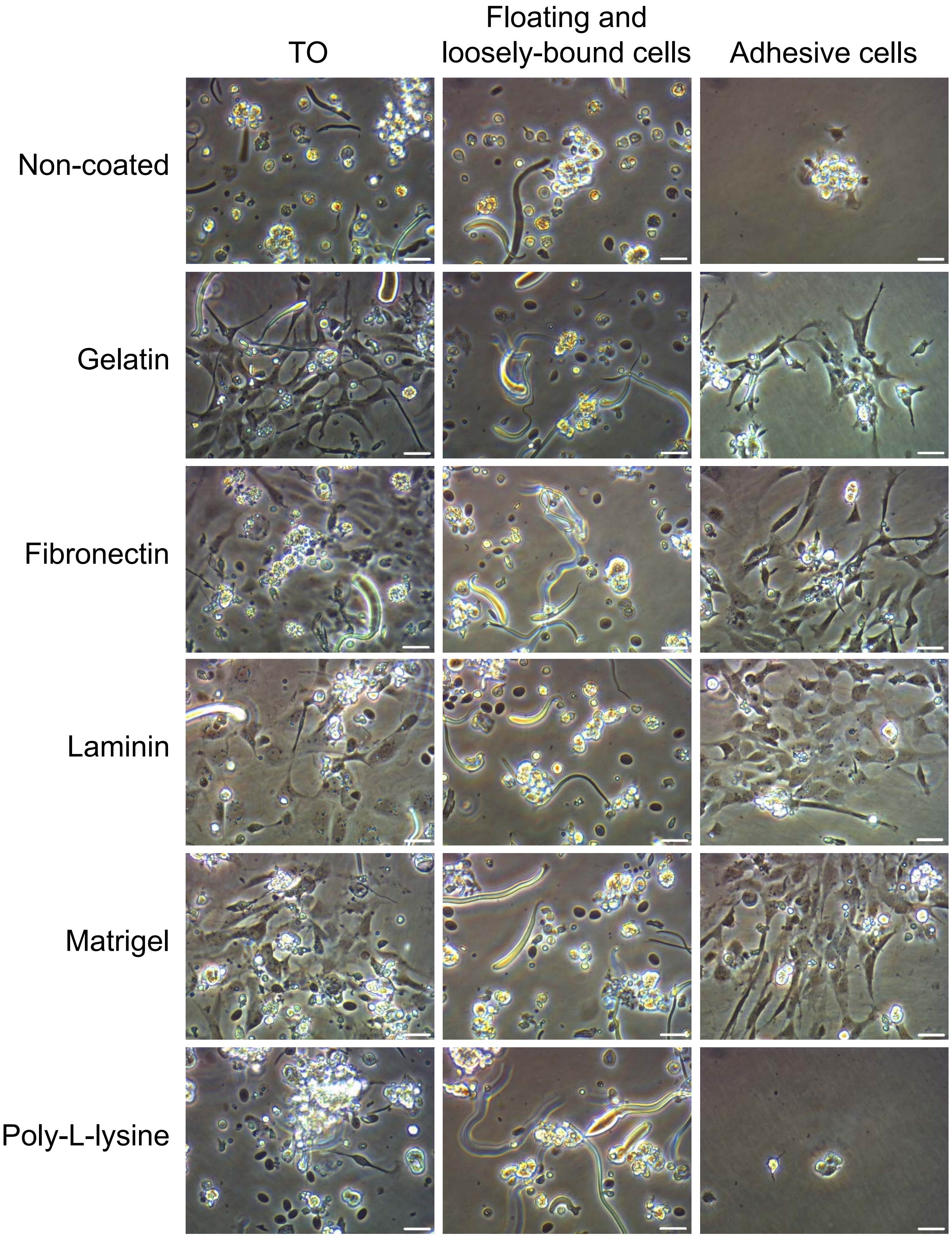

TO
Floating and loosely-bound cells
Adhesive cells
Non-coated
Gelatin
Fibronectin
Laminin
Matrigel
Poly-L-lysine
