# Supplementary material for: Enhanced Enrichment of Medaka Ovarian Germline Stem Cells by a Combination of Density Gradient Centrifugation and Differential Plating
Source: Biomolecules. 2020 Oct 24;10(11):1477. doi: 10.3390/biom10111477 (PMC7690863; doi:10.3390/biom10111477)
Supplement: Supplementary file 1 [file biomolecules-10-01477-s001.zip › biomolecules-945392-supplementary_final/Supplementary Figure S2_final.pdf]

Enhanced enrichment of medaka ovarian germline stem cells by a combination of density gradient centrifugation and differential plating

Jun Hyung Ryu, Seung Pyo Gong

Supplementary Figure S2. Examination of the optimal concentration of PKH26 for labeling the enriched ovarian cells. Crude ovarian cell population was collected from 10 adult females and enriched by a combination of Percoll density gradient centrifugation and differential plating. PKH26 was treated to the enriched ovarian cells with various concentrations for 3 min. (A) Percentage of fluorescent cells after PKH26 treatment. When the cells treated with more than 4 μM PKH26, significant increases of the percentage of fluorescent cells were observed compared to when the cells were treated with 0 and 2 μM PKH26. (B) Cell viability after PKH26 treatment. Cell viability was significantly decreased when the cells were treated with more than 8 μM PKH26. The values were expressed as mean±SD. [a-c]Different letters indicate significant differences, $P<0.05$. (C) Fluorescent images of the cells after PKH26 treatment. Fluorescent intensities were strong enough to progress further experiments when the cells were treated with more than 4 μM PKH26. Scale bar=100 μm.

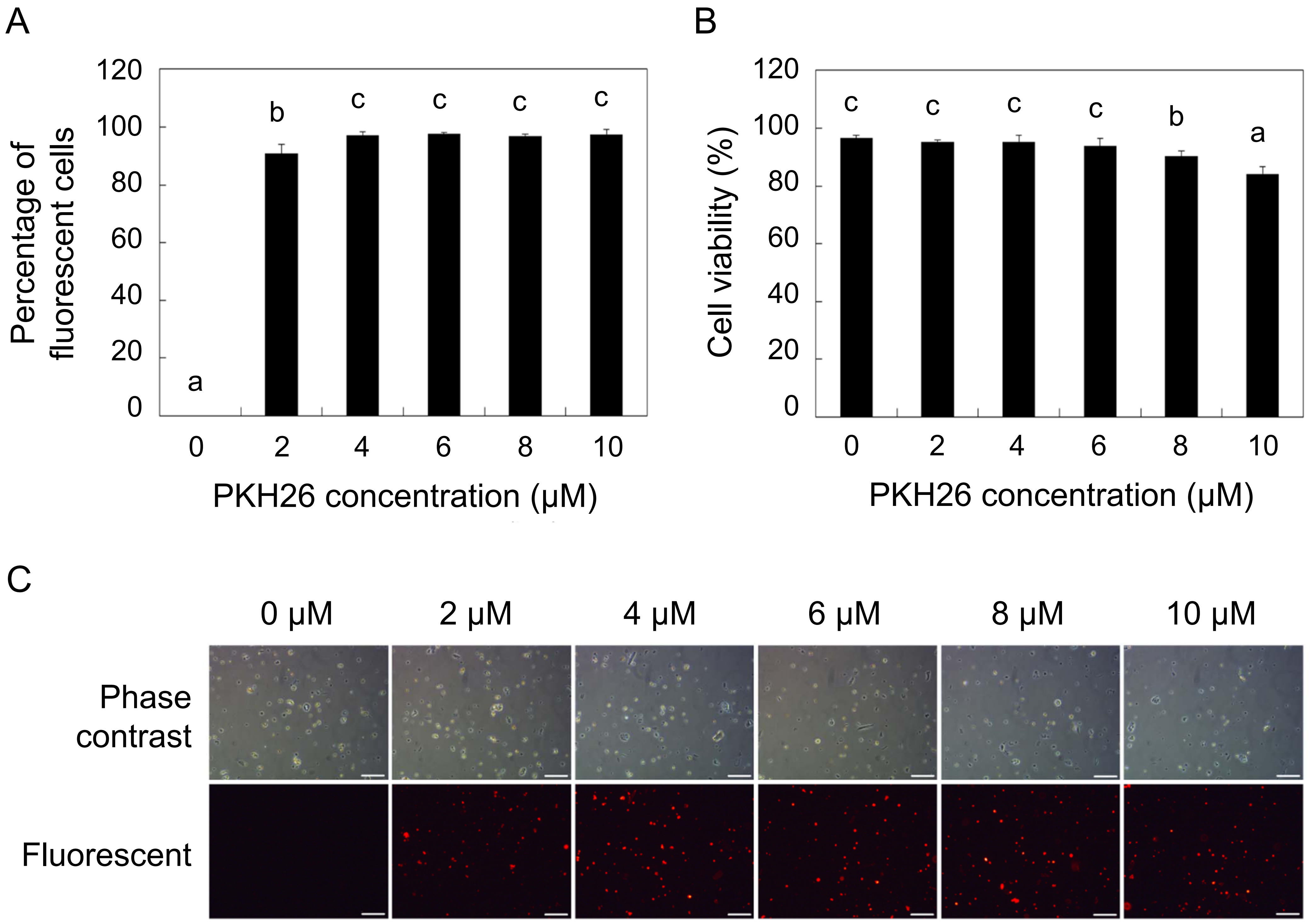
A
Percentage of fluorescent cells
120
100
80
60
40
20
0
a
b
c
c
c
c
0
2
4
6
8
10
PKH26 concentration (μM)
B
Cell viability (%)
120
100
80
60
40
20
0
c
c
c
c
b
a
0
2
4
6
8
10
PKH26 concentration (μM)
C
0 μM
2 μM
4 μM
6 μM
8 μM
10 μM
Phase contrast
Fluorescent
