# Supplementary material for: Enhanced Enrichment of Medaka Ovarian Germline Stem Cells by a Combination of Density Gradient Centrifugation and Differential Plating
Source: Biomolecules. 2020 Oct 24;10(11):1477. doi: 10.3390/biom10111477 (PMC7690863; doi:10.3390/biom10111477)
Supplement: Supplementary file 1 [file biomolecules-10-01477-s001.zip › biomolecules-945392-supplementary_final/Supplementary Figure S3_final.pdf]

Enhanced enrichment of medaka ovarian germline stem cells by a combination of density gradient centrifugation and differential plating

Jun Hyung Ryu, Seung Pyo Gong

Supplementary Figure S3. Wide angle pictures of Figure 6. Yellow dotted circles indicate the cells localized in gonadal region of the recipient larvae transplanted with total ovarian cells (TO) and the cells enriched by a combination of percoll density gradient centrifugation (PDGC) and differential plating (DP). White dotted areas indicate the cells located ectopically in posterior abdominal region of the recipient transplanted with embryonic cells (EC). Scale bar=200 μm.

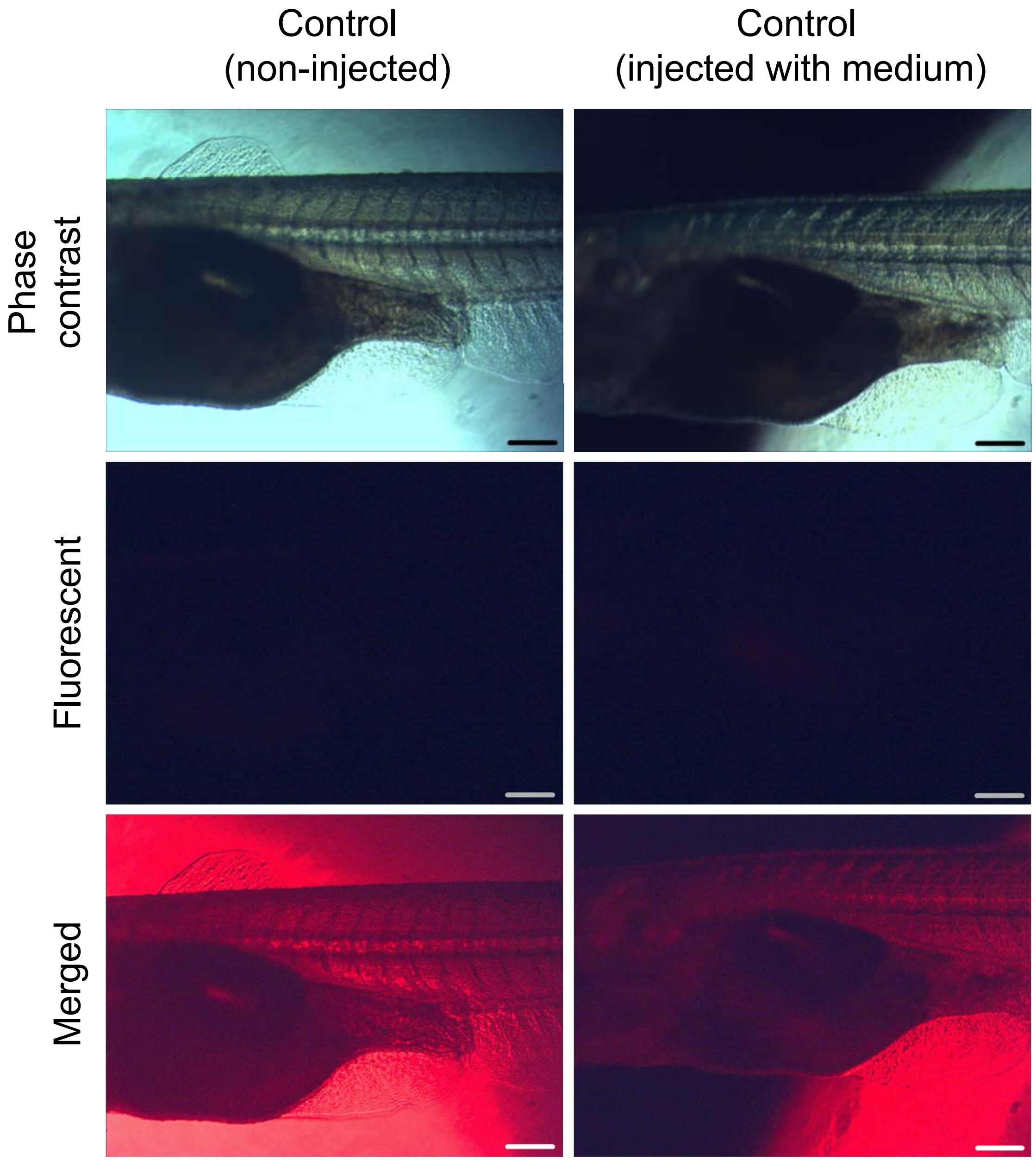

Control
(non-injected)
Control
(injected with medium)
Phase contrast
Fluorescent
Merged

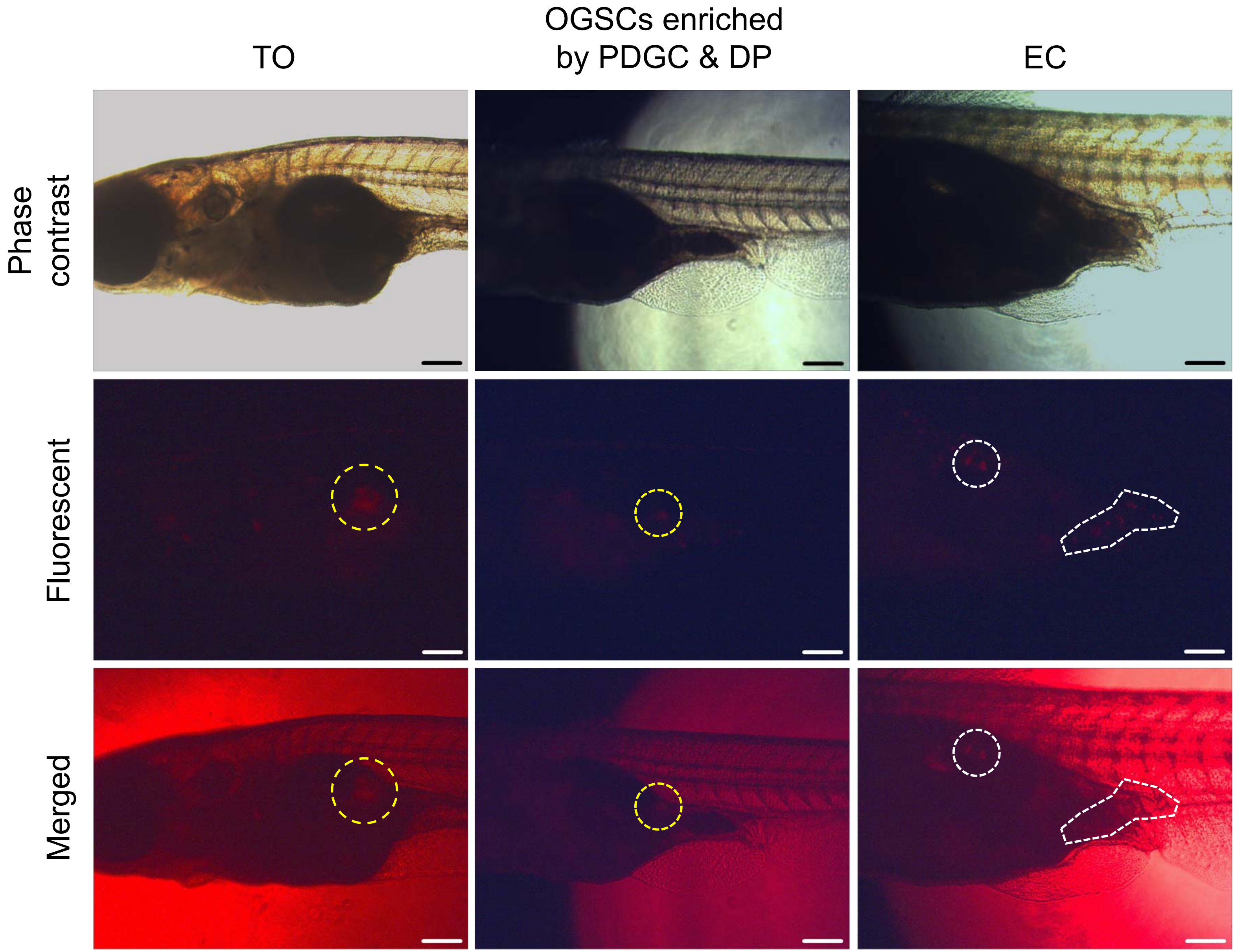
TO
OGSCs enriched
by PDGC & DP
EC
Phase
contrast
Fluorescent
Merged
